# Intelligence, Correspondence, &c.

**Published:** 1830-01-01

**Authors:** 


### INTELLIGENCE, CORRESPONDENCE, &c.

The Hospital Report of an interesting case, by Dr. Thwaites, is received, and will appear in the Clinical Review.

The offer from Edinburgh respecting the Review of a valuable publication lately issued from the Northern press, is declined, because the reviewer of the said work was already selected.

Many letters have been received during the last quarter on the business of the Journal, and which should have been privately answered, but the absence of the Editor on the Continent prevented punctuality. It is now too late to notice some of them, and Dr. J. will answer the others by post as soon as leisure permits.

Dr. ARMSTRONG.—It is to be particularly remembered that the notice of this lamented physician, which was drawn up in the course of one evening, makes no pretence to a biography—but merely to a sketch of Dr. Armstrong's professional character, with some reflexions on his short, but eventful life. Doubtless, some of his friends will give a more detailed account of the life and writings of that talented physician, whose premature death has left a widow and large family to deplore his loss.

*** We understand the Pupils of his Class are entering into a Subscription for a Bust, or other Memento, of their late Master.

We beg to inform Mr. Cowell, and several others, that the Journal cannot be forwarded to distant parts by the London publisher. It must be procured by the subscribers themselves, through the medium of their own booksellers.—The same answer to Mr. Wood, the American re-publisher.

MEDICAL PUPIL.

A Physician residing contiguous to the large Hospitals and Medical Schools at the West End has a Vacancy for a Pupil who may be entering the Profession or completing his studies in it.

*Terms:*—120 Guineas per annum, including the Practice of the Medical and Surgical Officers of an extensive Dispensary and ample opportunities of instruction in Midwifery. For Cards apply to the Editor of this Journal.

